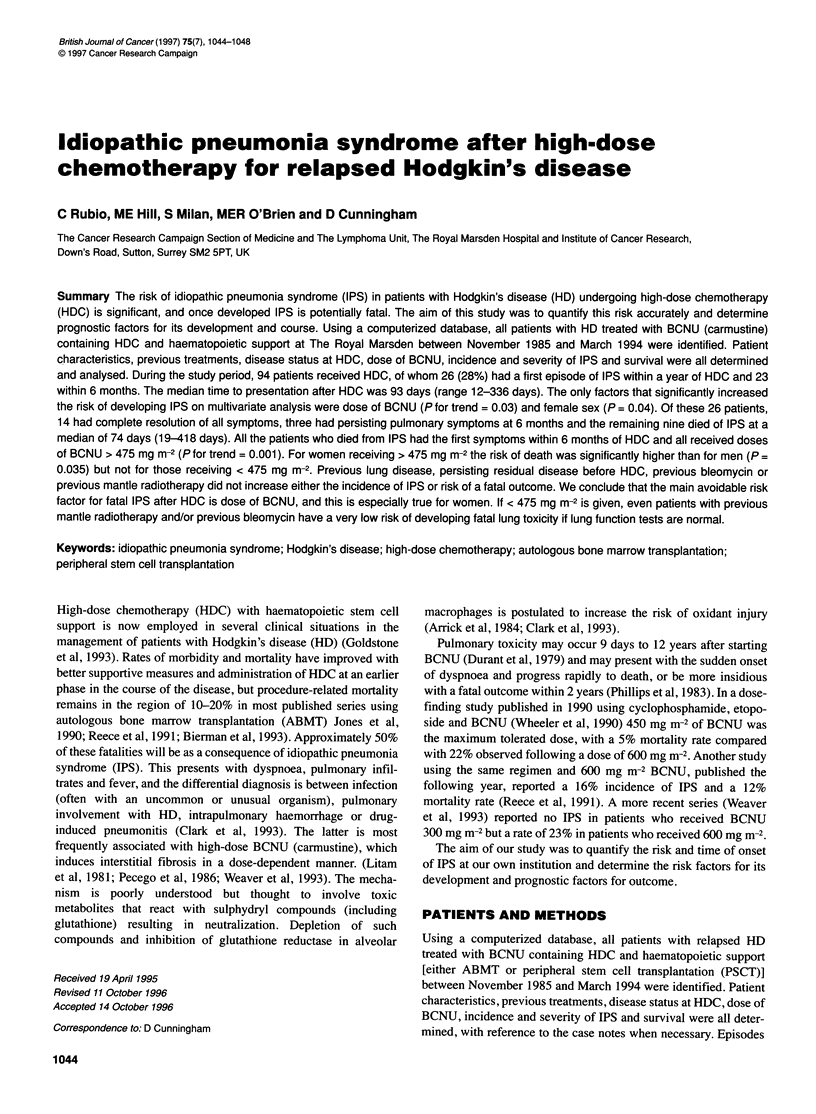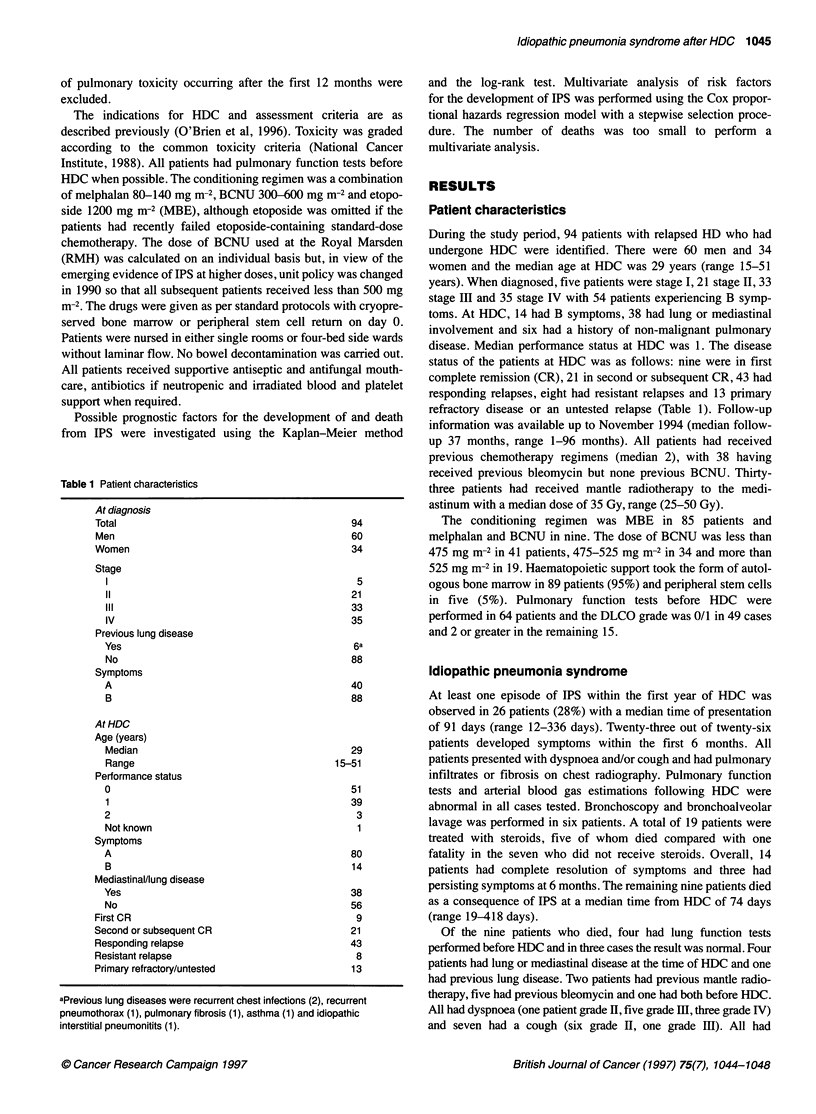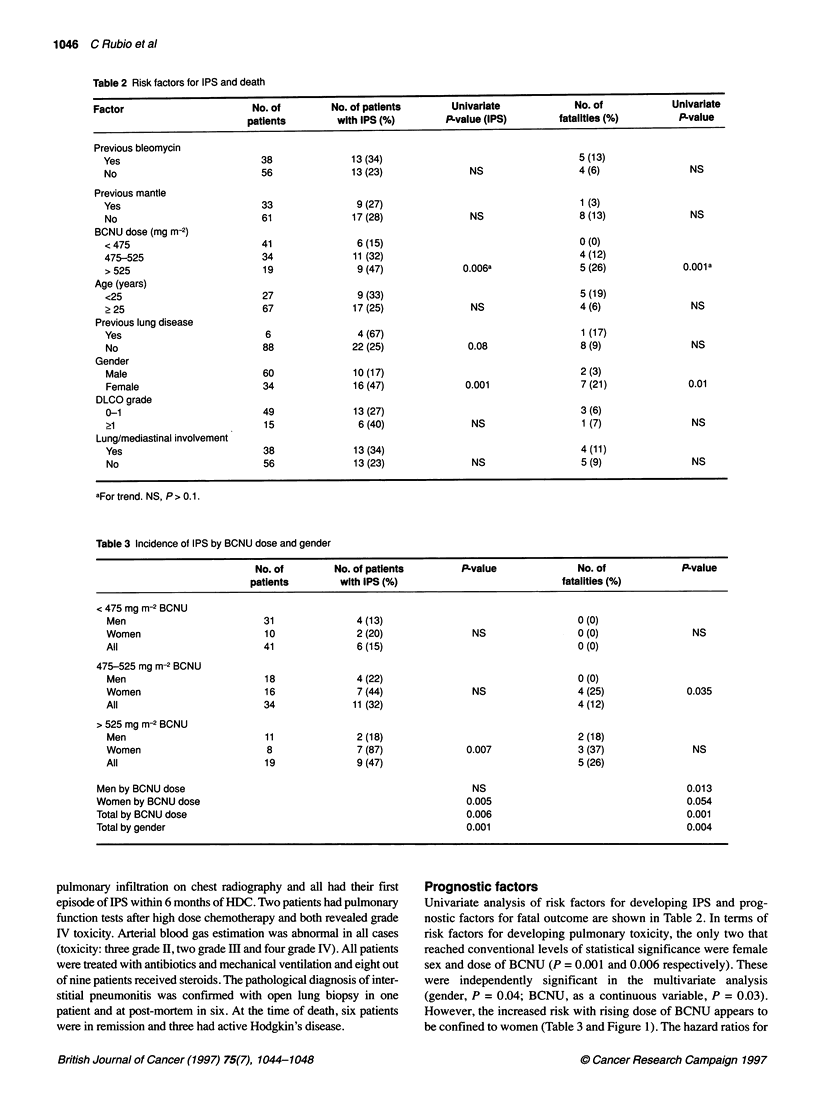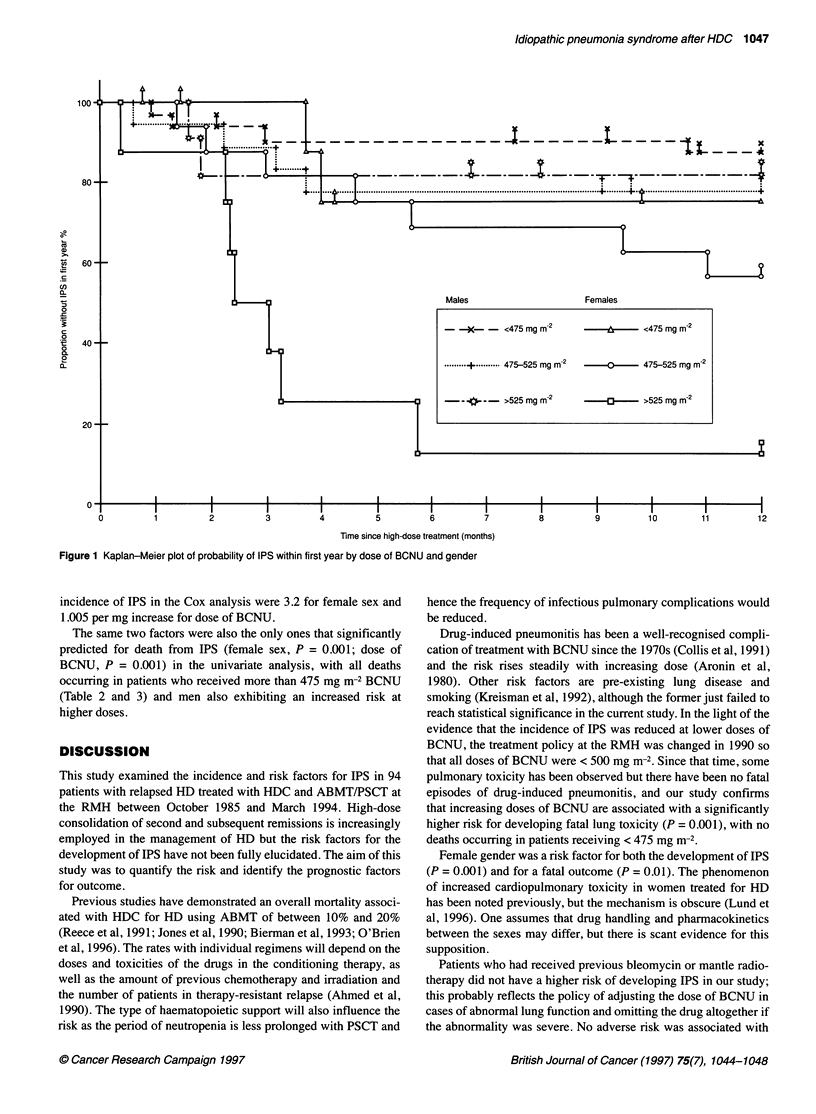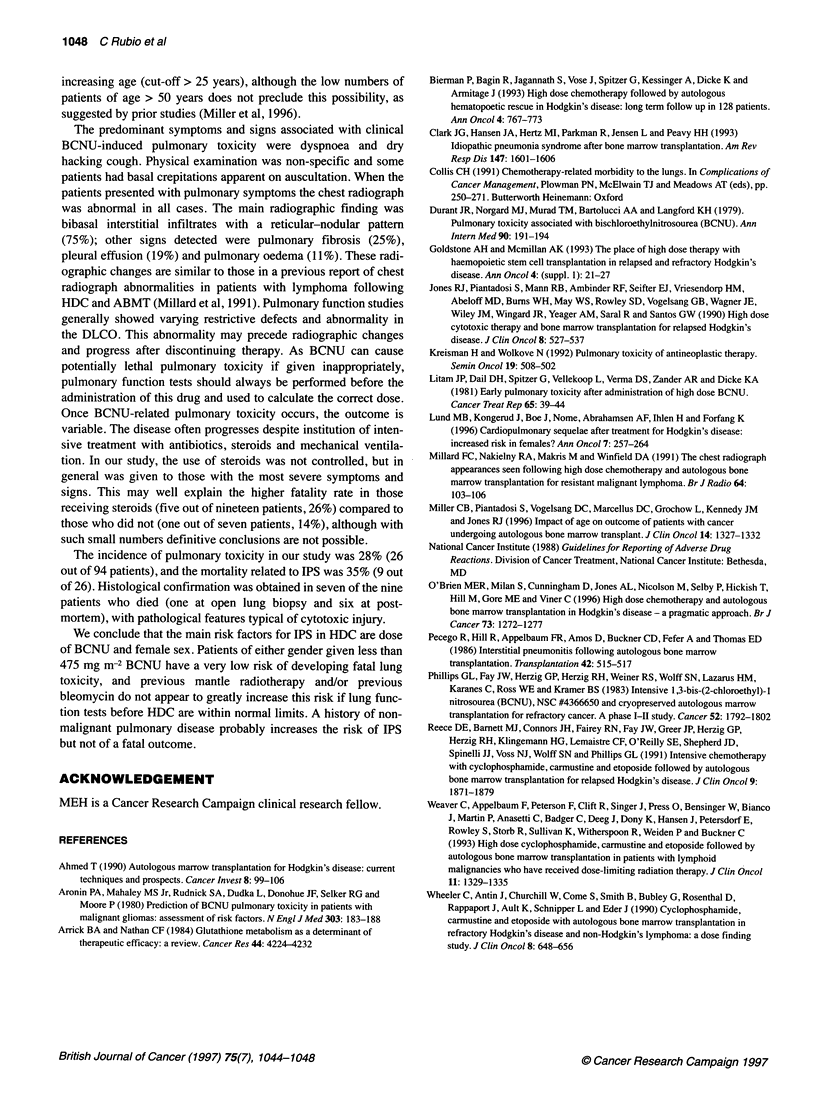# Idiopathic pneumonia syndrome after high-dose chemotherapy for relapsed Hodgkin's disease.

**DOI:** 10.1038/bjc.1997.178

**Published:** 1997

**Authors:** C. Rubio, M. E. Hill, S. Milan, M. E. O'Brien, D. Cunningham

**Affiliations:** The Cancer Research Campaign Section of Medicine, The Royal Marsden Hospital, Sutton, Surrey, UK.

## Abstract

The risk of idiopathic pneumonia syndrome (IPS) in patients with Hodgkin's disease (HD) undergoing high-dose chemotherapy (HDC) is significant, and once developed IPS is potentially fatal. The aim of this study was to quantify this risk accurately and determine prognostic factors for its development and course. Using a computerized database, all patients with HD treated with BCNU (carmustine) containing HDC and haematopoietic support at The Royal Marsden between November 1985 and March 1994 were identified. Patient characteristics, previous treatments, disease status at HDC, dose of BCNU, incidence and severity of IPS and survival were all determined and analysed. During the study period, 94 patients received HDC, of whom 26 (28%) had a first episode of IPS within a year of HDC and 23 within 6 months. The median time to presentation after HDC was 93 days (range 12-336 days). The only factors that significantly increased the risk of developing IPS on multivariate analysis were dose of BCNU (P for trend = 0.03) and female sex (P = 0.04). Of these 26 patients, 14 had complete resolution of all symptoms, three had persisting pulmonary symptoms at 6 months and the remaining nine died of IPS at a median of 74 days (19-418 days). All the patients who died from IPS had the first symptoms within 6 months of HDC and all received doses of BCNU > 475 mg m(-2) (P for trend = 0.001). For women receiving > 475 mg m(-2) the risk of death was significantly higher than for men (P = 0.035) but not for those receiving < 475 mg m(-2). Previous lung disease, persisting residual disease before HDC, previous bleomycin or previous mantle radiotherapy did not increase either the incidence of IPS or risk of a fatal outcome. We conclude that the main avoidable risk factor for fatal IPS after HDC is dose of BCNU, and this is especially true for women. If < 475 mg m(-2) is given, even patients with previous mantle radiotherapy and/or previous bleomycin have a very low risk of developing fatal lung toxicity if lung function tests are normal.


British Journal of Cancer (1997) 75(7), 1044–1048
© 1997 Cancer Research Campaign

### Idiopathic pneumonia syndrome after high-dose chemotherapy for relapsed Hodgkin's disease

**C Rubio, ME Hill, S Milan, MER O'Brien and D Cunningham**

The Cancer Research Campaign Section of Medicine and The Lymphoma Unit, The Royal Marsden Hospital and Institute of Cancer Research, Down's Road, Sutton, Surrey SM2 5PT, UK

**Summary** The risk of idiopathic pneumonia syndrome (IPS) in patients with Hodgkin's disease (HD) undergoing high-dose chemotherapy (HDC) is significant, and once developed IPS is potentially fatal. The aim of this study was to quantify this risk accurately and determine prognostic factors for its development and course. Using a computerized database, all patients with HD treated with BCNU (carmustine) containing HDC and haematopoietic support at The Royal Marsden between November 1985 and March 1994 were identified. Patient characteristics, previous treatments, disease status at HDC, dose of BCNU, incidence and severity of IPS and survival were all determined and analysed. During the study period, 94 patients received HDC, of whom 26 (28%) had a first episode of IPS within a year of HDC and 23 within 6 months. The median time to presentation after HDC was 93 days (range 12–336 days). The only factors that significantly increased the risk of developing IPS on multivariate analysis were dose of BCNU ($P$ for trend = 0.03) and female sex ($P$ = 0.04). Of these 26 patients, 14 had complete resolution of all symptoms, three had persisting pulmonary symptoms at 6 months and the remaining nine died of IPS at a median of 74 days (19–418 days). All the patients who died from IPS had the first symptoms within 6 months of HDC and all received doses of BCNU > 475 mg $m^{-2}$ ($P$ for trend = 0.001). For women receiving > 475 mg $m^{-2}$ the risk of death was significantly higher than for men ($P$ = 0.035) but not for those receiving < 475 mg $m^{-2}$. Previous lung disease, persisting residual disease before HDC, previous bleomycin or previous mantle radiotherapy did not increase either the incidence of IPS or risk of a fatal outcome. We conclude that the main avoidable risk factor for fatal IPS after HDC is dose of BCNU, and this is especially true for women. If < 475 mg $m^{-2}$ is given, even patients with previous mantle radiotherapy and/or previous bleomycin have a very low risk of developing fatal lung toxicity if lung function tests are normal.

**Keywords:** idiopathic pneumonia syndrome; Hodgkin's disease; high-dose chemotherapy; autologous bone marrow transplantation; peripheral stem cell transplantation

High-dose chemotherapy (HDC) with haematopoietic stem cell support is now employed in several clinical situations in the management of patients with Hodgkin's disease (HD) (Goldstone et al, 1993). Rates of morbidity and mortality have improved with better supportive measures and administration of HDC at an earlier phase in the course of the disease, but procedure-related mortality remains in the region of 10–20% in most published series using autologous bone marrow transplantation (ABMT) Jones et al, 1990; Reece et al, 1991; Bierman et al, 1993). Approximately 50% of these fatalities will be as a consequence of idiopathic pneumonia syndrome (IPS). This presents with dyspnoea, pulmonary infiltrates and fever, and the differential diagnosis is between infection (often with an uncommon or unusual organism), pulmonary involvement with HD, intrapulmonary haemorrhage or drug-induced pneumonitis (Clark et al, 1993). The latter is most frequently associated with high-dose BCNU (carmustine), which induces interstitial fibrosis in a dose-dependent manner. (Litam et al, 1981; Pecego et al, 1986; Weaver et al, 1993). The mechanism is poorly understood but thought to involve toxic metabolites that react with sulphydryl compounds (including glutathione) resulting in neutralization. Depletion of such compounds and inhibition of glutathione reductase in alveolar macrophages is postulated to increase the risk of oxidant injury (Arrick et al, 1984; Clark et al, 1993).

Pulmonary toxicity may occur 9 days to 12 years after starting BCNU (Durant et al, 1979) and may present with the sudden onset of dyspnoea and progress rapidly to death, or be more insidious with a fatal outcome within 2 years (Phillips et al, 1983). In a dose-finding study published in 1990 using cyclophosphamide, etoposide and BCNU (Wheeler et al, 1990) 450 mg $m^{-2}$ of BCNU was the maximum tolerated dose, with a 5% mortality rate compared with 22% observed following a dose of 600 mg $m^{-2}$. Another study using the same regimen and 600 mg $m^{-2}$ BCNU, published the following year, reported a 16% incidence of IPS and a 12% mortality rate (Reece et al, 1991). A more recent series (Weaver et al, 1993) reported no IPS in patients who received BCNU 300 mg $m^{-2}$ but a rate of 23% in patients who received 600 mg $m^{-2}$.

The aim of our study was to quantify the risk and time of onset of IPS at our own institution and determine the risk factors for its development and prognostic factors for outcome.

### PATIENTS AND METHODS

Using a computerized database, all patients with relapsed HD treated with BCNU containing HDC and haematopoietic support [either ABMT or peripheral stem cell transplantation (PSCT)] between November 1985 and March 1994 were identified. Patient characteristics, previous treatments, disease status at HDC, dose of BCNU, incidence and severity of IPS and survival were all determined, with reference to the case notes when necessary. Episodes

*Received 19 April 1995*
*Revised 11 October 1996*
*Accepted 14 October 1996*

*Correspondence to:* D Cunningham of pulmonary toxicity occurring after the first 12 months were excluded.

The indications for HDC and assessment criteria are as described previously (O'Brien et al, 1996). Toxicity was graded according to the common toxicity criteria (National Cancer Institute, 1988). All patients had pulmonary function tests before HDC when possible. The conditioning regimen was a combination of melphalan 80–140 mg $m^{-2}$, BCNU 300–600 mg $m^{-2}$ and etoposide 1200 mg $m^{-2}$ (MBE), although etoposide was omitted if the patients had recently failed etoposide-containing standard-dose chemotherapy. The dose of BCNU used at the Royal Marsden (RMH) was calculated on an individual basis but, in view of the emerging evidence of IPS at higher doses, unit policy was changed in 1990 so that all subsequent patients received less than 500 mg $m^{-2}$. The drugs were given as per standard protocols with cryopreserved bone marrow or peripheral stem cell return on day 0. Patients were nursed in either single rooms or four-bed side wards without laminar flow. No bowel decontamination was carried out. All patients received supportive antiseptic and antifungal mouthcare, antibiotics if neutropenic and irradiated blood and platelet support when required.

Possible prognostic factors for the development of and death from IPS were investigated using the Kaplan–Meier method and the log-rank test. Multivariate analysis of risk factors for the development of IPS was performed using the Cox proportional hazards regression model with a stepwise selection procedure. The number of deaths was too small to perform a multivariate analysis.

**Table 1** Patient characteristics

| | |
|---|---|
| *At diagnosis* | |
| Total | 94 |
| Men | 60 |
| Women | 34 |
| Stage | |
| I | 5 |
| II | 21 |
| III | 33 |
| IV | 35 |
| Previous lung disease | |
| Yes | 6[a] |
| No | 88 |
| Symptoms | |
| A | 40 |
| B | 88 |
| *At HDC* | |
| Age (years) | |
| Median | 29 |
| Range | 15–51 |
| Performance status | |
| 0 | 51 |
| 1 | 39 |
| 2 | 3 |
| Not known | 1 |
| Symptoms | |
| A | 80 |
| B | 14 |
| Mediastinal/lung disease | |
| Yes | 38 |
| No | 56 |
| First CR | 9 |
| Second or subsequent CR | 21 |
| Responding relapse | 43 |
| Resistant relapse | 8 |
| Primary refractory/untested | 13 |

[a]Previous lung diseases were recurrent chest infections (2), recurrent pneumothorax (1), pulmonary fibrosis (1), asthma (1) and idiopathic interstitial pneumonitits (1).

### RESULTS

### Patient characteristics

During the study period, 94 patients with relapsed HD who had undergone HDC were identified. There were 60 men and 34 women and the median age at HDC was 29 years (range 15–51 years). When diagnosed, five patients were stage I, 21 stage II, 33 stage III and 35 stage IV with 54 patients experiencing B symptoms. At HDC, 14 had B symptoms, 38 had lung or mediastinal involvement and six had a history of non-malignant pulmonary disease. Median performance status at HDC was 1. The disease status of the patients at HDC was as follows: nine were in first complete remission (CR), 21 in second or subsequent CR, 43 had responding relapses, eight had resistant relapses and 13 primary refractory disease or an untested relapse (Table 1). Follow-up information was available up to November 1994 (median follow-up 37 months, range 1–96 months). All patients had received previous chemotherapy regimens (median 2), with 38 having received previous bleomycin but none previous BCNU. Thirty-three patients had received mantle radiotherapy to the mediastinum with a median dose of 35 Gy, range (25–50 Gy).

The conditioning regimen was MBE in 85 patients and melphalan and BCNU in nine. The dose of BCNU was less than 475 mg $m^{-2}$ in 41 patients, 475–525 mg $m^{-2}$ in 34 and more than 525 mg $m^{-2}$ in 19. Haematopoietic support took the form of autologous bone marrow in 89 patients (95%) and peripheral stem cells in five (5%). Pulmonary function tests before HDC were performed in 64 patients and the DLCO grade was 0/1 in 49 cases and 2 or greater in the remaining 15.

### Idiopathic pneumonia syndrome

At least one episode of IPS within the first year of HDC was observed in 26 patients (28%) with a median time of presentation of 91 days (range 12–336 days). Twenty-three out of twenty-six patients developed symptoms within the first 6 months. All patients presented with dyspnoea and/or cough and had pulmonary infiltrates or fibrosis on chest radiography. Pulmonary function tests and arterial blood gas estimations following HDC were abnormal in all cases tested. Bronchoscopy and bronchoalveolar lavage was performed in six patients. A total of 19 patients were treated with steroids, five of whom died compared with one fatality in the seven who did not receive steroids. Overall, 14 patients had complete resolution of symptoms and three had persisting symptoms at 6 months. The remaining nine patients died as a consequence of IPS at a median time from HDC of 74 days (range 19–418 days).

Of the nine patients who died, four had lung function tests performed before HDC and in three cases the result was normal. Four patients had lung or mediastinal disease at the time of HDC and one had previous lung disease. Two patients had previous mantle radiotherapy, five had previous bleomycin and one had both before HDC. All had dyspnoea (one patient grade II, five grade III, three grade IV) and seven had a cough (six grade II, one grade III). All had

© Cancer Research Campaign 1997

**Table 2** Risk factors for IPS and death

| Factor | No. of patients | No. of patients with IPS (%) | Univariate P-value (IPS) | No. of fatalities (%) | Univariate P-value |
|---|---|---|---|---|---|
| Previous bleomycin | | | | | |
| Yes | 38 | 13 (34) | | 5 (13) | |
| No | 56 | 13 (23) | NS | 4 (6) | NS |
| Previous mantle | | | | | |
| Yes | 33 | 9 (27) | | 1 (3) | |
| No | 61 | 17 (28) | NS | 8 (13) | NS |
| BCNU dose (mg $m^{-2}$) | | | | | |
| < 475 | 41 | 6 (15) | | 0 (0) | |
| 475–525 | 34 | 11 (32) | | 4 (12) | |
| > 525 | 19 | 9 (47) | 0.006[a] | 5 (26) | 0.001[a] |
| Age (years) | | | | | |
| <25 | 27 | 9 (33) | | 5 (19) | |
| ≥ 25 | 67 | 17 (25) | NS | 4 (6) | NS |
| Previous lung disease | | | | | |
| Yes | 6 | 4 (67) | | 1 (17) | |
| No | 88 | 22 (25) | 0.08 | 8 (9) | NS |
| Gender | | | | | |
| Male | 60 | 10 (17) | | 2 (3) | |
| Female | 34 | 16 (47) | 0.001 | 7 (21) | 0.01 |
| DLCO grade | | | | | |
| 0–1 | 49 | 13 (27) | | 3 (6) | |
| ≥1 | 15 | 6 (40) | NS | 1 (7) | NS |
| Lung/mediastinal involvement | | | | | |
| Yes | 38 | 13 (34) | | 4 (11) | |
| No | 56 | 13 (23) | NS | 5 (9) | NS |

[a]For trend. NS, $P > 0.1$.

**Table 3** Incidence of IPS by BCNU dose and gender

| | No. of patients | No. of patients with IPS (%) | P-value | No. of fatalities (%) | P-value |
|---|---|---|---|---|---|
| < 475 mg $m^{-2}$ BCNU | | | | | |
| Men | 31 | 4 (13) | | 0 (0) | |
| Women | 10 | 2 (20) | NS | 0 (0) | NS |
| All | 41 | 6 (15) | | 0 (0) | |
| 475–525 mg $m^{-2}$ BCNU | | | | | |
| Men | 18 | 4 (22) | | 0 (0) | |
| Women | 16 | 7 (44) | NS | 4 (25) | 0.035 |
| All | 34 | 11 (32) | | 4 (12) | |
| > 525 mg $m^{-2}$ BCNU | | | | | |
| Men | 11 | 2 (18) | | 2 (18) | |
| Women | 8 | 7 (87) | 0.007 | 3 (37) | NS |
| All | 19 | 9 (47) | | 5 (26) | |
| Men by BCNU dose | | | NS | | 0.013 |
| Women by BCNU dose | | | 0.005 | | 0.054 |
| Total by BCNU dose | | | 0.006 | | 0.001 |
| Total by gender | | | 0.001 | | 0.004 |

pulmonary infiltration on chest radiography and all had their first episode of IPS within 6 months of HDC. Two patients had pulmonary function tests after high dose chemotherapy and both revealed grade IV toxicity. Arterial blood gas estimation was abnormal in all cases (toxicity: three grade II, two grade III and four grade IV). All patients were treated with antibiotics and mechanical ventilation and eight out of nine patients received steroids. The pathological diagnosis of interstitial pneumonitis was confirmed with open lung biopsy in one patient and at post-mortem in six. At the time of death, six patients were in remission and three had active Hodgkin's disease.

### Prognostic factors

Univariate analysis of risk factors for developing IPS and prognostic factors for fatal outcome are shown in Table 2. In terms of risk factors for developing pulmonary toxicity, the only two that reached conventional levels of statistical significance were female sex and dose of BCNU ($P = 0.001$ and 0.006 respectively). These were independently significant in the multivariate analysis (gender, $P = 0.04$; BCNU, as a continuous variable, $P = 0.03$). However, the increased risk with rising dose of BCNU appears to be confined to women (Table 3 and Figure 1). The hazard ratios for

© Cancer Research Campaign 1997

**Figure 1** Kaplan–Meier plot of probability of IPS within first year by dose of BCNU and gender incidence of IPS in the Cox analysis were 3.2 for female sex and 1.005 per mg increase for dose of BCNU.

The same two factors were also the only ones that significantly predicted for death from IPS (female sex, $P$ = 0.001; dose of BCNU, $P$ = 0.001) in the univariate analysis, with all deaths occurring in patients who received more than 475 mg $m^{-2}$ BCNU (Table 2 and 3) and men also exhibiting an increased risk at higher doses.

### DISCUSSION

This study examined the incidence and risk factors for IPS in 94 patients with relapsed HD treated with HDC and ABMT/PSCT at the RMH between October 1985 and March 1994. High-dose consolidation of second and subsequent remissions is increasingly employed in the management of HD but the risk factors for the development of IPS have not been fully elucidated. The aim of this study was to quantify the risk and identify the prognostic factors for outcome.

Previous studies have demonstrated an overall mortality associated with HDC for HD using ABMT of between 10% and 20% (Reece et al, 1991; Jones et al, 1990; Bierman et al, 1993; O'Brien et al, 1996). The rates with individual regimens will depend on the doses and toxicities of the drugs in the conditioning therapy, as well as the amount of previous chemotherapy and irradiation and the number of patients in therapy-resistant relapse (Ahmed et al, 1990). The type of haematopoietic support will also influence the risk as the period of neutropenia is less prolonged with PSCT and hence the frequency of infectious pulmonary complications would be reduced.

Drug-induced pneumonitis has been a well-recognised complication of treatment with BCNU since the 1970s (Collis et al, 1991) and the risk rises steadily with increasing dose (Aronin et al, 1980). Other risk factors are pre-existing lung disease and smoking (Kreisman et al, 1992), although the former just failed to reach statistical significance in the current study. In the light of the evidence that the incidence of IPS was reduced at lower doses of BCNU, the treatment policy at the RMH was changed in 1990 so that all doses of BCNU were < 500 mg $m^{-2}$. Since that time, some pulmonary toxicity has been observed but there have been no fatal episodes of drug-induced pneumonitis, and our study confirms that increasing doses of BCNU are associated with a significantly higher risk for developing fatal lung toxicity ($P$ = 0.001), with no deaths occurring in patients receiving < 475 mg $m^{-2}$.

Female gender was a risk factor for both the development of IPS ($P$ = 0.001) and for a fatal outcome ($P$ = 0.01). The phenomenon of increased cardiopulmonary toxicity in women treated for HD has been noted previously, but the mechanism is obscure (Lund et al, 1996). One assumes that drug handling and pharmacokinetics between the sexes may differ, but there is scant evidence for this supposition.

Patients who had received previous bleomycin or mantle radiotherapy did not have a higher risk of developing IPS in our study; this probably reflects the policy of adjusting the dose of BCNU in cases of abnormal lung function and omitting the drug altogether if the abnormality was severe. No adverse risk was associated with increasing age (cut-off > 25 years), although the low numbers of patients of age > 50 years does not preclude this possibility, as suggested by prior studies (Miller et al, 1996).

The predominant symptoms and signs associated with clinical BCNU-induced pulmonary toxicity were dyspnoea and dry hacking cough. Physical examination was non-specific and some patients had basal crepitations apparent on auscultation. When the patients presented with pulmonary symptoms the chest radiograph was abnormal in all cases. The main radiographic finding was bibasal interstitial infiltrates with a reticular–nodular pattern (75%); other signs detected were pulmonary fibrosis (25%), pleural effusion (19%) and pulmonary oedema (11%). These radiographic changes are similar to those in a previous report of chest radiograph abnormalities in patients with lymphoma following HDC and ABMT (Millard et al, 1991). Pulmonary function studies generally showed varying restrictive defects and abnormality in the DLCO. This abnormality may precede radiographic changes and progress after discontinuing therapy. As BCNU can cause potentially lethal pulmonary toxicity if given inappropriately, pulmonary function tests should always be performed before the administration of this drug and used to calculate the correct dose. Once BCNU-related pulmonary toxicity occurs, the outcome is variable. The disease often progresses despite institution of intensive treatment with antibiotics, steroids and mechanical ventilation. In our study, the use of steroids was not controlled, but in general was given to those with the most severe symptoms and signs. This may well explain the higher fatality rate in those receiving steroids (five out of nineteen patients, 26%) compared to those who did not (one out of seven patients, 14%), although with such small numbers definitive conclusions are not possible.

The incidence of pulmonary toxicity in our study was 28% (26 out of 94 patients), and the mortality related to IPS was 35% (9 out of 26). Histological confirmation was obtained in seven of the nine patients who died (one at open lung biopsy and six at post-mortem), with pathological features typical of cytotoxic injury.

We conclude that the main risk factors for IPS in HDC are dose of BCNU and female sex. Patients of either gender given less than 475 mg $m^{-2}$ BCNU have a very low risk of developing fatal lung toxicity, and previous mantle radiotherapy and/or previous bleomycin do not appear to greatly increase this risk if lung function tests before HDC are within normal limits. A history of non-malignant pulmonary disease probably increases the risk of IPS but not of a fatal outcome.

### ACKNOWLEDGEMENT

MEH is a Cancer Research Campaign clinical research fellow.

### REFERENCES

Ahmed T (1990) Autologous marrow transplantation for Hodgkin's disease: current techniques and prospects. *Cancer Invest* **8**: 99–106

Aronin PA, Mahaley MS Jr, Rudnick SA, Dudka L, Donohue JF, Selker RG and Moore P (1980) Prediction of BCNU pulmonary toxicity in patients with malignant gliomas: assessment of risk factors. *N Engl J Med* **303**: 183–188

Arrick BA and Nathan CF (1984) Glutathione metabolism as a determinant of therapeutic efficacy: a review. *Cancer Res* **44**: 4224–4232

Bierman P, Bagin R, Jagannath S, Vose J, Spitzer G, Kessinger A, Dicke K and Armitage J (1993) High dose chemotherapy followed by autologous hematopoetic rescue in Hodgkin's disease: long term follow up in 128 patients. *Ann Oncol* **4**: 767–773

Clark JG, Hansen JA, Hertz MI, Parkman R, Jensen L and Peavy HH (1993) Idiopathic pneumonia syndrome after bone marrow transplantation. *Am Rev Resp Dis* **147**: 1601–1606

Collis CH (1991) Chemotherapy-related morbidity to the lungs. In *Complications of Cancer Management*, Plowman PN, McElwain TJ and Meadows AT (eds), pp. 250–271. Butterworth Heinemann: Oxford

Durant JR, Norgard MJ, Murad TM, Bartolucci AA and Langford KH (1979). Pulmonary toxicity associated with bischloroethylnitrosourea (BCNU). *Ann Intern Med* **90**: 191–194

Goldstone AH and Mcmillan AK (1993) The place of high dose therapy with haemopoietic stem cell transplantation in relapsed and refractory Hodgkin's disease. *Ann Oncol* **4**: (suppl. 1): 21–27

Jones RJ, Piantadosi S, Mann RB, Ambinder RF, Seifter EJ, Vriesendorp HM, Abeloff MD, Burns WH, May WS, Rowley SD, Vogelsang GB, Wagner JE, Wiley JM, Wingard JR, Yeager AM, Saral R and Santos GW (1990) High dose cytotoxic therapy and bone marrow transplantation for relapsed Hodgkin's disease. *J Clin Oncol* **8**: 527–537

Kreisman H and Wolkove N (1992) Pulmonary toxicity of antineoplastic therapy. *Semin Oncol* **19**: 508–502

Litam JP, Dail DH, Spitzer G, Vellekoop L, Verma DS, Zander AR and Dicke KA (1981) Early pulmonary toxicity after administration of high dose BCNU. *Cancer Treat Rep* **65**: 39–44

Lund MB, Kongerud J, Boe J, Nome, Abrahamsen AF, Ihlen H and Forfang K (1996) Cardiopulmonary sequelae after treatment for Hodgkin's disease: increased risk in females? *Ann Oncol* **7**: 257–264

Millard FC, Nakielny RA, Makris M and Winfield DA (1991) The chest radiograph appearances seen following high dose chemotherapy and autologous bone marrow transplantation for resistant malignant lymphoma. *Br J Radio* **64**: 103–106

Miller CB, Piantadosi S, Vogelsang DC, Marcellus DC, Grochow L, Kennedy JM and Jones RJ (1996) Impact of age on outcome of patients with cancer undergoing autologous bone marrow transplant. *J Clin Oncol* **14**: 1327–1332

National Cancer Institute (1988) *Guidelines for Reporting of Adverse Drug Reactions*. Division of Cancer Treatment, National Cancer Institute: Bethesda, MD

O'Brien MER, Milan S, Cunningham D, Jones AL, Nicolson M, Selby P, Hickish T, Hill M, Gore ME and Viner C (1996) High dose chemotherapy and autologous bone marrow transplantation in Hodgkin's disease – a pragmatic approach. *Br J Cancer* **73**: 1272–1277

Pecego R, Hill R, Appelbaum FR, Amos D, Buckner CD, Fefer A and Thomas ED (1986) Interstitial pneumonitis following autologous bone marrow transplantation. *Transplantation* **42**: 515–517

Phillips GL, Fay JW, Herzig GP, Herzig RH, Weiner RS, Wolff SN, Lazarus HM, Karanes C, Ross WE and Kramer BS (1983) Intensive 1,3-bis-(2-chloroethyl)-1 nitrosourea (BCNU), NSC #4366650 and cryopreserved autologous marrow transplantation for refractory cancer. A phase I–II study. *Cancer* **52**: 1792–1802

Reece DE, Barnett MJ, Connors JH, Fairey RN, Fay JW, Greer JP, Herzig GP, Herzig RH, Klingemann HG, Lemaistre CF, O'Reilly SE, Shepherd JD, Spinelli JJ, Voss NJ, Wolff SN and Phillips GL (1991) Intensive chemotherapy with cyclophosphamide, carmustine and etoposide followed by autologous bone marrow transplantation for relapsed Hodgkin's disease. *J Clin Oncol* **9**: 1871–1879

Weaver C, Appelbaum F, Peterson F, Clift R, Singer J, Press O, Bensinger W, Bianco J, Martin P, Anasetti C, Badger C, Deeg J, Dony K, Hansen J, Petersdorf E, Rowley S, Storb R, Sullivan K, Witherspoon R, Weiden P and Buckner C (1993) High dose cyclophosphamide, carmustine and etoposide followed by autologous bone marrow transplantation in patients with lymphoid malignancies who have received dose-limiting radiation therapy. *J Clin Oncol* **11**: 1329–1335

Wheeler C, Antin J, Churchill W, Come S, Smith B, Bubley G, Rosenthal D, Rappaport J, Ault K, Schnipper L and Eder J (1990) Cyclophosphamide, carmustine and etoposide with autologous bone marrow transplantation in refractory Hodgkin's disease and non-Hodgkin's lymphoma: a dose finding study. *J Clin Oncol* **8**: 648–656

© Cancer Research Campaign 1997